# MicroRNA-210-3p Promotes Chondrogenic Differentiation and Inhibits Adipogenic Differentiation Correlated with HIF-3*α* Signalling in Bone Marrow Mesenchymal Stem Cells

**DOI:** 10.1155/2021/6699910

**Published:** 2021-04-10

**Authors:** Meng Yang, Xin Yan, Fu-Zhen Yuan, Jing Ye, Ming-Ze Du, Zi-Mu Mao, Bing-Bing Xu, You-Rong Chen, Yi-Fan Song, Bao-Shi Fan, Jia-Kuo Yu

**Affiliations:** ^1^Sports Medicine Department, Beijing Key Laboratory of Sports Injuries, Peking University Third Hospital, Beijing, China; ^2^Institute of Sports Medicine of Peking University, Beijing, China; ^3^School of Clinical Medicine, Weifang Medical University, Weifang, China

## Abstract

Cartilage injury of the knee joint is very common. Due to the limited self-healing ability of articular cartilage, osteoarthritis is very likely to occur if left untreated. Bone marrow mesenchymal stem cells (BMMSCs) are widely used in the study of cartilage injury due to their low immunity and good amplification ability, but they still have disadvantages, such as heterogeneous undifferentiated cells. MicroRNAs can regulate the chondrogenic differentiation ability of MSCs by inhibiting or promoting mRNA translation and degradation. In this research, we primarily investigated the effect of microRNA-210-3p (miR-210-3p) on chondrogenic and adipogenic differentiation of BMMSCs in vitro. Our results demonstrate that miR-210-3p promoted chondrogenic differentiation and inhibited adipogenic differentiation of rat BMMSCs, which was related to the HIF-3*α* signalling pathway. Additionally, miR-210-3p promotes mRNA and protein levels of the chondrogenic expression genes COLII and SOX9 and inhibits mRNA and protein levels of the adipogenic expression genes PPAR*γ* and LPL. Thus, miR-210-3p combined with BMMSCs is a candidate for future clinical applications in cartilage regeneration and could represent a promising new therapeutic target for OA.

## 1. Introduction

Articular cartilage injury caused by trauma and arthritic diseases is one of the main causes of disability in middle-aged and elderly people [1]. Due to the lack of vascular and nerve structures in articular cartilage, once an injury occurs, it usually does not heal well [2]. There are many ways to treat articular cartilage injury, and mesenchymal stem cells (MSCs) are widely studied due to their low immunogenicity, rapid amplification, and multidirectional differentiation. Bone marrow-derived MSCs constitute the best candidate for bone and cartilage tissue engineering [3], although they are composed of adipose tissue [4], peripheral blood [5], umbilical cord [6], and other tissue sources. There are drawbacks of MSC transplantation as well; for example, MSC transplantation might lead to dissimilar nondifferentiation and differentiation of populations [7]. Therefore, new strategies to enhance the differentiation of MSCs into specific chondrocytes remain to be proposed.

MicroRNAs (miRNAs) are a small class of noncoding single-stranded RNAs that are regulated at the posttranscriptional level by inhibiting mRNA translation or promoting mRNA degradation [8]. Many experimental data have indicated that miRNAs play a crucial regulatory role in the chondrogenic differentiation of MSCs [9–11]. By the regulation of specific genes, several miRNAs (miR-145 [12], miR-365 [13], and miR-218 [14]) have been shown to modulate chondrogenic differentiation of MSCs. miR-210 has been extensively studied and has many confirmed functional targets, such as cell proliferation, DNA damage response, angiogenesis, and apoptosis [15–17]. However, the participation of miR-210-3p in BMMSC chondrogenic differentiation remains unclear. There is a delicate balance between chondrogenic differentiation and adipogenic differentiation of BMMSCs. Therefore, we investigated the effect of miR-210-3p on MSC chondrogenic and adipogenic differentiation.

## 2. Materials and Methods

### 2.1. Cell Culture

The Animal Ethics Committee of the Third Hospital of Peking University approved all animal experiments. Ten Sprague-Dawley male rats weighing between 100 and 120 g at 6 weeks of age were used in this study. Rats were euthanized by CO_2_ inhalation, the hind leg soft tissue was rapidly removed, and the femurs were preserved in phosphate-buffered saline (PBS). BMMSCs were collected and cultured in minimal essential medium (*α*-MEM; Gibco) with 10% foetal bovine serum (FBS; Gibco) in an environment of 37°C and 5% CO_2_. When cells were 80%–90% confluent, 0.25% trypsin was digested and passed at a 1 : 2 ratio. Third-generation purified BMMSCs were used in the experiment.

### 2.2. Detection of BMMSC Surface Markers

Following cell counting, the concentration of mononuclear cells (MNCs) in the bone marrow was adjusted to 10^7^ cells/ml by the addition of PBS. A total of 100 *μ*l was placed into a 1.5 ml EP tube. Concomitantly, monocolour and bicolour isotype control groups were prepared. Antibody staining for the flow cytometry assay was conducted as follows: CD44-FITC (Biosciences, USA), CD90-FITC (BioLegend, USA), CD34-PE (GeneTex, USA), and CD45-FITC (Bioss, China) were incubated at dark room temperature for 30 min (PE (phycoerythrin), FITC (fluorescein isothiocyanate), and CD (cluster of differentiation)). Samples were rinsed twice with PBS, resuspended in 400 *μ*l PBS, and finally added to the flow tubes for flow cytometry detection (Biosciences, USA).

### 2.3. Cell Transfection

BMMSCs were inoculated into 6-well plates at a density of 2 × 10^5^ one day before transfection. To improve transfection efficiency, cells were cultured in 2% FBS for 12 h after cell adherence. Then, 50 nM miR-210-3p mimics, 100 nM miR-210-3p inhibitors (RiboBio, China), and HIF-3*α* siRNA (RiboBio, China) were transfected into MSCs using Lipofectamine 2000 (Invitrogen, USA) according to the manufacturers' instructions. Control cells did not receive any transfection reagent and were referred to as the normal group. Quantitative real-time reverse transcription-polymerization chain reaction (qRT-PCR) was used to select the optimal transfection concentration for subsequent experiments. Cells were harvested after 48 h for qRT-PCR or 72 h for Western blot analysis.

### 2.4. Chondrogenic Differentiation of BMMSCs

To further identify the chondrogenic differentiation potential of BMMSCs, third-generation BMMSCs were collected for 1 × 10^5^/well density vaccination in six-well plates. When cells reached 40%~60% confluence, miR-210 mimics and inhibitors were separately transfected into MSCs. The three groups of cells were digested and counted after 36 hours, and 3 × 10^5^ cells were transferred into a 15 ml centrifuge tube. Induction was performed according to the instructions of chondrogenic differentiation medium (Cyagen Biosciences, China). When cells clustered together, the bottom of the centrifuge tube was lightly flicked to dislodge the pellets from the bottom of the tube and suspend it in the liquid. BMMSC induction was performed for 21 days, and the culture medium was changed every 3 days. To enhance the transfection efficiency, miR-210 mimics and inhibitors were transfected again on days 7 and 14 of induction. After fixing the pellets and paraffin-embedded sections, staining for chondrogenic differentiation was performed using Alcian blue solution (Cyagen Biosciences, China) for 1 h at room temperature.

### 2.5. Adipogenic Differentiation of BMMSCs

Third-generation BMMSCs were collected and vaccinated at a density of 1 × 10^5^/well into six-well plates. When cells reached 60%~80% confluence, miR-210-3p mimics and inhibitors were separately transfected into BMMSCs. At the end of transfection, adipogenic differentiation medium was used for induction. The medium replacement was performed in accordance with the instructions. Staining was performed 7 days after induction. Staining for adipogenic differentiation was performed using Oil Red O solution for 20 min. Differentiation media and staining fluid were purchased from Cyagen Biosciences (China).

### 2.6. qRT-PCR

TRIzol reagent (Invitrogen, USA) was used to extract total RNA from cells. One microgram of RNA was transcribed into cDNA using a RevertAid first-strand cDNA synthesis kit (Thermo Fisher Scientific, USA), and SYBR Green Master Mix (Thermo Fisher Scientific, USA) was used for qRT-PCR. Chondrogenic marker genes SOX9 and COLII, adipogenic marker genes PPAR*γ* and LPL, and miRNA-210-3p and HIF-3*α* gene levels were detected; and GAPDH and U6 were used as internal controls. Relative quantification analysis was performed using the 1/2^*ΔΔ*CT^ method.

### 2.7. Western Blot Analysis

Cells transfected into each group for 72 h were collected and lysed on ice with 4 *μ*l benzenesulfonyl fluoride (PMSF) mixed with 200 *μ*l radioimmunoprecipitation (RIPA) for 30 min. After centrifugation and extraction of the supernatant, the BCA kit was used to determine protein concentration. Proteins (30 *μ*l per group) were electrophoretically transferred to PVDF membranes. Primary antibodies against GAPDH (1 : 10000, Abcam, UK), SOX9 (1 : 5000, Abcam, UK), COLII (1 : 3000, Abcam, UK), PPAR*γ* (1 : 1000, Abcam, UK), LPL (1 : 5000, Abcam, UK), and HIF-3*α* (1 : 2000, Abcam, UK) were incubated for 10 h at 4°C. Membranes were then incubated with the secondary antibody (1 : 10000, Abcam, UK) for 2 h at 4°C.

### 2.8. Statistical Analysis

SPSS 25.0 was used for statistical analysis. Experimental values are expressed as the mean ± standard deviation (SD). Significant differences among groups were evaluated by the *t*-test. *P* < 0.05 is considered statistically significant.

## 3. Results

### 3.1. Characterization of BMMSCs and miR-210 Transfection

**Figure 1:**
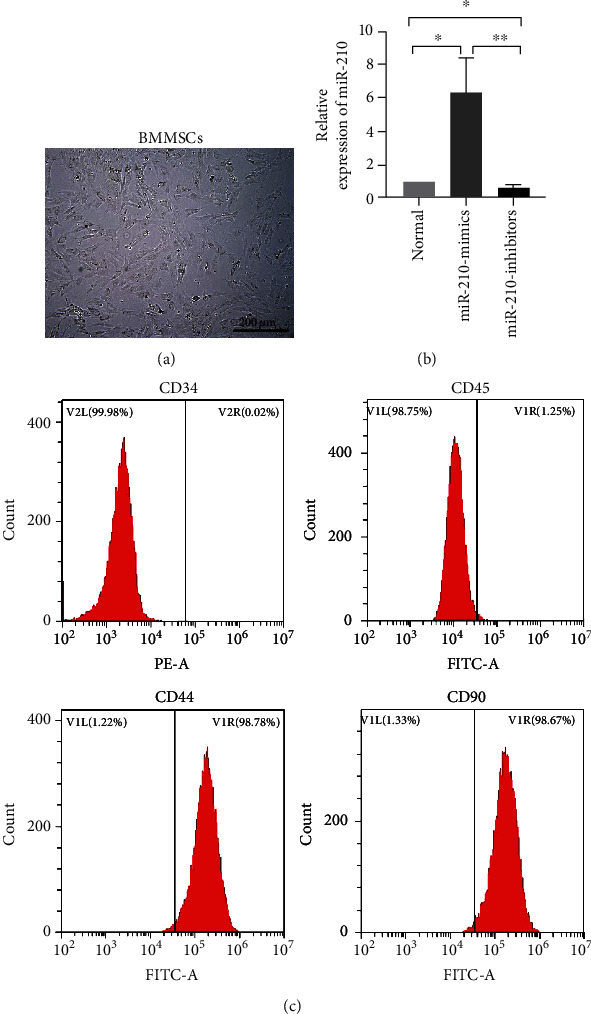
Characterization of BMMSCs. (a) Morphological characteristics of adherent cells from bone marrow at passage 3 under an inverted phase-contrast microscope. (b) miRNA levels of miR-210 between three groups were evaluated by qRT-PCR. (c) Flow cytometry analysis of BMMSCs was positive for CD44 and CD90 and negative for CD34 and CD45.

BMMSCs exhibited a homogeneous monolayer of fibroblasts adhering to the plastic (Figure 1(a)). The transfection efficiency of miR-210 mimics and inhibitors is shown in Figure 1(b). To verify whether our cultured BMMSCs exhibit MSC characteristics, the cells were analysed using flow cytometry. The results showed positive expression of CD44 and CD90 and negative expression of CD34 and CD45 (Figure 1(c)), consistent with flow-mediated identification of MSCs.

### 3.2. miR-210 Promotes Chondrogenic Differentiation in BMMSCs

**Figure 2:**
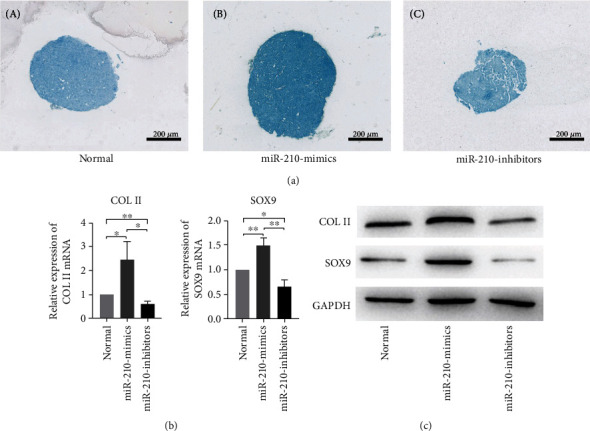
miR-210 promotes BMMSC chondrogenic differentiation. (a) Alcian blue staining results induced by chondrogenic medium in the (A) normal, (B) miR-210 mimic, and (C) miR-210 inhibitor groups. (b) mRNA levels of COLII and SOX9 were evaluated by qRT-PCR. All statistical tests were based on the NC group. (c) Western blot analysis of COLII and SOX9 protein expression in the normal, miR-210 mimic, and miR-210 inhibitor groups. GAPDH was the internal control (^∗^*P* < 0.05, ^∗∗^*P* < 0.01).

As shown in Figures 2(a) and 2(b), miR-210 mimics promoted an increase in the extracellular matrix and deepened Alcian blue staining. In contrast (Figure 2(a), C), miR-210 inhibitors reduced the extracellular matrix. In addition, this group of pellets was more likely to break. To further investigate whether miR-210 induces chondrogenic differentiation of BMMSCs, expression levels of the relevant classical chondrogenic marker genes COLII and SOX9 were detected by qRT-PCR. qRT-PCR showed that transfection with miR-210 mimics resulted in an increase in the mRNA expression of chondrocyte markers, which were significantly downregulated in BMMSCs transfected with miR-210 inhibitors (Figure 2(b)). To confirm that mRNA expression leads to protein expression, we assessed expression levels of various proteins involved in chondrogenic differentiation. As shown in Figure 2(c), the miR-210 mimic group exhibited increased expression levels of COLII and SOX9 in BMMSCs. These results suggest that miR-210-3p may promote differentiation of BMMSCs into chondrocytes in vitro.

### 3.3. miR-210 Inhibits Adipogenic Differentiation in BMMSCs

**Figure 3:**
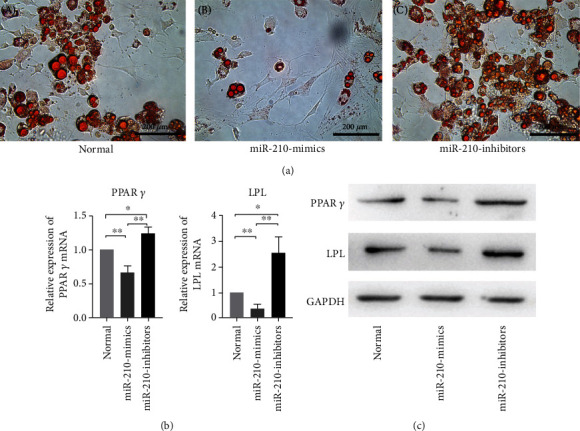
miR-210 inhibits BMMSC adipogenic differentiation. (a) Oil Red O staining results of lipid droplets induced by adipogenic medium in (A) normal, (B) miR-210 mimic, and (C) miR-210 inhibitor groups. (b) mRNA levels of PPAR*γ* and LPL were evaluated by qRT-PCR. All statistical tests were based on the normal group. (c) Western blot analysis of PPAR*γ* and LPL protein expression in the normal, miR-210 mimic, and miR-210 inhibitor groups. GAPDH was the internal control (^∗^*P* < 0.05, ^∗∗^*P* < 0.01).

miR-210 mimics inhibit BMMSC lipid droplets, and miR-210 inhibitors promote lipid droplet formation. This suggests that overexpression of miR-210 suppresses adipogenic differentiation in BMMSCs (Figure 3(a), B). Expression levels of the adipogenic marker genes PPAR*γ* and LPL were examined by qRT-PCR. Results showed that transfection of the miR-210 mimic resulted in a significant reduction in adipogenic marker mRNA, whereas these markers were significantly increased in miR-210 inhibitor-transfected BMMSCs (Figure 3(b)). To verify that mRNA expression leads to protein expression, we measured expression levels of classical proteins involved in adipogenic differentiation. As shown in Figure 3(c), the miR-210 mimic group exhibited reduced PPAR and LPL expression levels in BMMSCs. These results suggest that miR-210 inhibits the differentiation of BMMSC adipogenesis in vitro.

### 3.4. miR-210 Directly Targets HIF-3*α*

**Figure 4:**
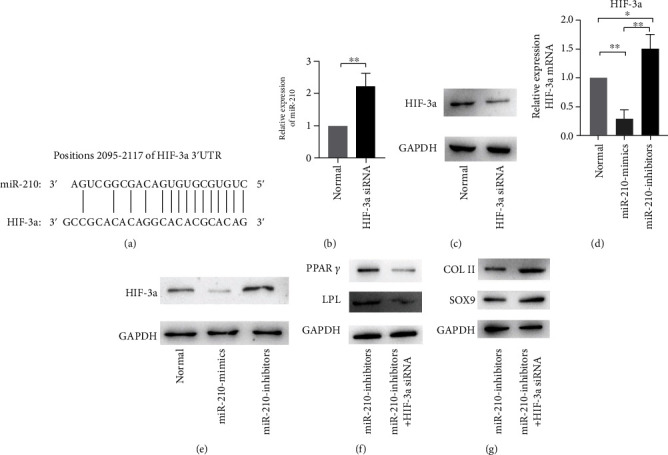
miR-210 directly regulates HIF-3*α*. (a) Binding sites of miR-210-3p and HIF-3*α*. (b) miR-210 mRNA levels were measured after transfection with HIF-3*α* siRNA or the control group. (c) HIF-3*α* protein levels were measured after transfection with HIF-3*α* siRNA or the control group. GAPDH was the internal control. (d) HIF-3*α* mRNA levels were measured after transfection of the three groups. (e) HIF-3*α* protein levels were measured after transfection with normal, miR-210 mimic, and miR-210 inhibitor groups. (f) PPAR*γ* and LPL protein levels were measured after transfection with the miR-210 inhibitor group and the miR-210 inhibitor+HIF-3*α* siRNA group. (g) COLII and SOX9 protein levels were measured after transfection with the miR-210 inhibitor group and the miR-210 inhibitor+HIF-3*α* siRNA group (^∗^*P* < 0.05, ^∗∗^*P* < 0.01).

**Figure 5:**
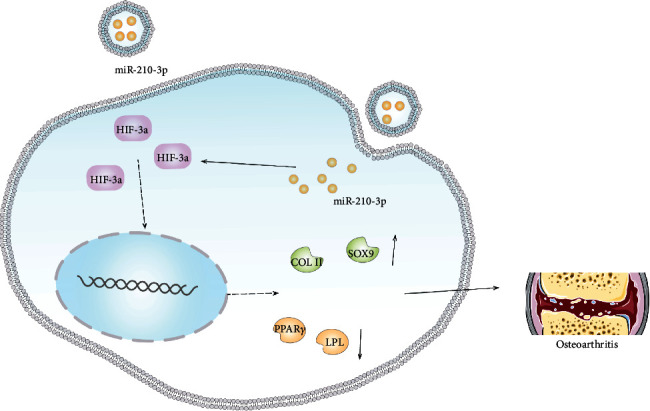
The effects of miR-210-3p on chondrogenesis and adipogenesis of BMMSCs. The combination of miR-210-3p with BMMSCs may represent the right choice for the application of cartilage regeneration in osteoarthritis.

The TargetScan database (http://www.targetscan.org) predicted that there was a miR-210-3p binding site in the 3′UTR of HIF-3*α* (position 2095-2117; Figure 4(a)). To analyse predicted target genes of miRNA-mRNA interactions, we knocked down HIF-3*α*. The HIF-3*α* siRNA transfection results showed that the miR-210′ mRNA expression (Figure 4(b)) in the BMMSCs was significantly increased after transfection. The HIF-3*α* siRNA transfection results showed that HIF-3*α*′ protein expression (Figure 4(c)) in BMMSCs was significantly decreased after transfection. In addition, the results showed that transfection of the miR-210 mimic group reduced mRNA (Figure 4(d)) and protein expression (Figure 4(e)) of HIF-3*α*. Markway et al.'s results showed that HIF-3*α* knockdown promoted an enhanced chondrogenic phenotype [18], so we believed that miR-210-3p might promote the chondrogenic ability of BMMSCs by directly targeting the HIF-3*α* pathway. Results of the cotransfection of miR-210 inhibitors and HIF-3*α* siRNA and the transfection of miR-210 inhibitors alone showed that expression of PPAR*γ* and LPL in the cotransfection group was decreased (Figure 4(f)). This indicated that the miR-210 inhibitor group promoted adipogenesis of MSCs by directly targeting and decreasing expression of the HIF-3*α* gene. Results of the cotransfection of miR-210 inhibitors and HIF-3*α* siRNA and the transfection of miR-210 inhibitors alone showed that the expression of COLII and SOX9 in the cotransfection group was increased (Figure 4(g)). This indicated that the miR-210 inhibitor group reduced chondrogenesis in MSCs by directly targeting and increasing the expression of the HIF-3*α* gene. Once the expression of the HIF-3*α* gene was knocked down, miR-210 inhibitors could not inhibit the chondrogenesis of MSCs. To illustrate the effect of miR-210-3p on chondrogenesis and adipogenesis formation of BMMSCs more vividly, we drew a pattern diagram (Figure 5). According to the pattern diagram, miR-210-3p promotes chondrogenic differentiation and inhibits adipogenic differentiation of BMMSCs by promoting chondrogenic expression of COLII and SOX9 genes and inhibiting adipogenic expression of PPAR*γ* and LPL genes. Therefore, the combination of miR-210-3p with BMMSCs may be the right choice for the application of cartilage regeneration in osteoarthritis.

## 4. Discussion

There are two kinds of miR-210 (miR-210-3p, miR-210-5p). miR-210-3p is the guidance chain integrated into RISC, while miR-210-5p is the passenger chain deactivated by degradation [8]. Recent results have shown that miR-210 is highly expressed in most tumours. It has been associated with essential targets for cancer and the cell cycle, metabolism, cell survival, and many other functions [15–17].

In this study, we examined how miR-210 regulates chondrogenic differentiation of BMMSCs. We found that miR-210 mimics actively promoted chondrogenic differentiation of BMMSCs. In contrast, miR-210 inhibitors suppressed chondrogenic differentiation of BMMSCs. It is well known that there is a delicate balance between chondrogenic differentiation and adipogenic differentiation in BMMSCs. Therefore, we studied the effects of miR-210-3p on adipogenic differentiation. Results showed that miR-210 mimics inhibited adipogenic differentiation of BMMSCs and that miR-210 inhibitors promoted adipogenic differentiation, indicating that miR-210 can bind with BMMSCs to promote BMMSC chondrogenic transformation and inhibit adipogenic transformation. Thus, miR-210 combined with BMMSCs may be the right candidate for future clinical applications in osteochondral regeneration and could be a promising new therapeutic target for OA.

We suggest that miR-210-3p may promote chondrogenesis and inhibit adipogenesis of BMMSCs by directly targeting HIF-3*α*. The results of Jia et al. [19], Kai et al. [20] and Li et al. [21] all indicated that HIF-3*α* was the direct target of miR-210. Their results were consistent with our results, and increased miR-210 reduced the expression of HIF-3*α*. In addition, Markway et al.'s [18] study showed that HIF-3*α* expression is associated with the chondrocyte phenotype, and excessive expression of HIF-3*α* induced hypertrophy of chondrocytes. This is consistent with our conclusion that miR-210 inhibits expression of the HIF-3*α* gene and promotes cartilage formation. Moreover, the results of Li et al. [22] showed that miR-210 promoted adipogenic differentiation of mesenchymal stem cells, which was consistent with our conclusion. In addition, BMMSCs can differentiate into three lineages. Our experiment investigated the ability of miR-210 stem cells to differentiate into chondrogenic and adipogenic lineages. Many studies have demonstrated that miR-210 promotes osteogenic differentiation of stem cells [22–25]. Notably, the results of Li et al. [22] demonstrated that miR-210 promoted osteogenic differentiation of rat bone marrow stem cells. The results of Li et al.'s [22] study also demonstrated that miR-210-3p promoted osteogenic differentiation correlated with Wnt signalling in rBMSCs.

In conclusion, our results demonstrated that overexpression of miR-210-3p enhanced the expression of cartilage markers and promoted early chondrogenic differentiation of BMMSCs. Furthermore, our results showed that miR-210-3p reduces the expression of adipogenesis markers. In addition, miR-210-3p may regulate BMMSC chondrogenesis and adipogenesis via the miR-210-3p/HIF-3*α* pathway. Hence, miR-210-3p might represent a therapeutic strategy when applied to cartilage-related diseases using BMMSC-based therapies.

## Data Availability

The data used to support the results of the study are contained in this article.
